# Speech After Long Silence—An Appraisee-Based Comprehensive Analysis With Retrospective and Future Perspectives on Current ID Policy of Transpersons in China

**DOI:** 10.3389/fpubh.2021.793162

**Published:** 2021-11-30

**Authors:** Jian Shi, Yadong Guo, María José Cavagnaro, Jifeng Cai, Zhuoying Liu

**Affiliations:** ^1^Department of Hematology and Critical Care Medicine, The Third Xiangya Hospital, Central South University, Changsha, China; ^2^Department of Spine Surgery, The Third Xiangya Hospital, Central South University, Changsha, China; ^3^Department of Forensic Science, School of Basic Medical Sciences, Central South University, Changsha, China; ^4^College of Medicine-Phoenix, University of Arizona, Phoenix, AZ, United States; ^5^Health Law Research Center, School of Law, Central South University, Changsha, China

**Keywords:** transpersons, public health, ID policy, psychosexual health, anti-transgender prejudice, health law

## Abstract

As the sexual minority in China, transpersons remain faced with various realistic challenges. In recent years, however, there has been a significant progress made in the protection given to the rights that transpersons deserve. Currently, the citizens who have changed their gender through sex reassignment surgery can make applications to the local police station for changing their gender registration and get issued a new ID card. This is regarded as a crucial milestone in reducing the bias against transpersons and protecting their legitimate rights in China. Highlighted by the case of an extraordinary appraisee who have received SRS to change from male to female and started a new life with a new ID, not only does this article construe the current ID policy and the detailed process of ID card change for transpersons in China, it also reveals the living and developmental conditions facing transpersons in China. Finally, the visibility of the community of transpersons is improved to eradicate the discrimination against transpersons.

## Introduction

Jin Xing, a Chinese transgender celebrity, once said “If the world is a sea, the heterosexual is a land, the homosexual is an island, but what I choose is a stone that can be placed on the land or the island, but neither of them belongs to me.” (“I Can I BB” Qipa Shuo/Season 2, 20150711, iQIYI, Inc.). In China, the government takes a vague stance on lesbians, gays, bisexuals, transgender, and intersex (LGBTI). As one of the most populous countries around the world, China provides a significant base of the world's largest LGBTI community (1, 2). Despite a huge number of LGBTI in China (an estimate of 50–70 million population according to the seventh national population census of China), both state and local governments have chosen to distance themselves from those social issues in relation to sexual orientation and gender identity, holding a neutral attitude (3). The civil rights to which LGBTI people are entitled are exempt from protection in many parts of the country. For example, sex identification, marriage, kindred, employment, social welfare and others (4–6). Up to now, LGBTI still suffer discrimination from those who consider it evil or contradictory to Chinese traditional culture, with transpersons in particular. In general, transgender can be classed into multiple categories. Besides, there is still no unified statement regarding the types of transsexuality in the academic circle. In this paper, our discussion is limited to those transpersons who have received sex-reassignment surgery (SRS).

Currently, there are some countries permitting their citizens to change their gender. Among them, Sweden took the lead in allowing people to change their legal gender identity in as early as 1972 (7). Since then, legal authorization has been increasingly expanded thanks to the bravery and dedication of social activists, which has prompted more and more other countries to follow suit in the formulation and enforcement of sex/gender identity recognition laws. The implications of the laws are made diverse and specific. For example, the United States, Italy, Japan, South Korea and Canada give recognition to the change in sex/gender in line with medical standards. In comparison, the United Kingdom, German, and Spanish Federal Constitutional Court jurisprudence apply the self-identity standards of recognition given to sex/gender change (8).

In China, transpersons encounter the toughest challenges, for example, they are subjected to unfair treatment and discrimination when their gender identity is made known to others (9). It is common for those male-to-female people to enter into such professions as sex workers, entertainers, and performers. The female-to-male community is deemed less acceptable (10). In addition, medical professionals often lack willingness to serve transpersons, who are left with limited access to medical care. Transpersons suffer discrimination and unfair treatment in medical facilities. Unexpectedly, more progress has been made in the protection of transsexual's rights than that of homosexuals, with the change in sex/gender identity receiving official recognition (11). As for the policies related to transpersons, in 2008, the Bureau of Public Security granted approval to household registration (known as Hu Kou in Chinese) after SRS. The citizens who have changed their gender identity through SRS can apply to the local police station for changing the gender registration, with the gender identification issued by the domestic tertiary hospital, the public certification issued by the notarial department, or the certification issued by judicial authentication institution. After approval is granted from the higher departments of the Bureau of Public Security, the local public security police station deals with gender-change registration through the formalities. The gender-change registration, with all the formalities gone through, can be completed in the police station of the seat of registered permanent residence rapidly. Then, the applicant would be issued the new ID cards and have updated household registrations.

It marks the first time that transpersons receive official recognition. Therefore, it is a legitimate right for Chinese transpersons to change their gender on ID cards and household registrations. In spite of this, it is still not the case that all transpersons going through full sex-reassignment surgery (SRS) are entitled to make the change. Notably, there are few successful cases of transpersons receiving a new ID since the policy came into force. On the one hand, most people attach much importance to personal privacy. On the other hand, these social issues attract little attention from appraisal institutions. Therefore, there remain some questions needing to be answered. How has the policy actually been enforced in China? Can transpersons be successful in getting a new ID with a different gender identity after surgery? Fortunately, a case was collected from our cooperative appraisal institution, which is conducive to understanding the current state of transpersons. Let's take a look at the following case.

## Case Presentation and Appraisal Opinions

An appraisee, Xiao Ming (pseudonym), received SRS in September 2018 in Thailand. In order to change the gender identity as shown on household registration from male to female, Xiao Ming was given gender identification and offered judicial appraisal opinions in line with the rules set out by the Public Security Register Department. With approval granted by the local police department in charge of household registration, Xiao Ming hired an appraisal institution to carry out identification of her gender in March 2019. All the information presented in this study was collected from her and the cooperative appraisal institution.

Initially, Xiao Ming made application to an appraisal institution, along with three notarial certificates received from the notary offices. Notarization affairs: the translation of the medical record in the Thai language. The medical certificate was issued by a hospital in Thailand, the purpose of which is to certify that Xiao Ming has received a sex reassignment surgery performed by a registered doctor.

Examination results: there was no evidence suggesting psychosis. Diagnosis: gender anxiety disorder and gender identity disorder. Changing from male to female, Xiao Ming received SRS in Thailand. Besides, Xiao Ming received the surgeries purposed to reduce laryngeal prominence and correct breast. These operations were completed smoothly. Currently, the health condition is stable. Then, the appraiser carried out forensic examination on Xiao Ming, which confirmed that the appraisee was generally healthy. The appraisee was fully conscious and dressed as a female. Physical examination: no beard on the upper lip, no distinctive laryngeal prominence on the neck. With double plump female breasts, arc operation scars are visible at the bottom edge of double breasts. Perineal examination: no penile or scrotal structures. Labium majus, minus, and vagina can be seen after resetting surgery. The analysis and description of the appraisal constitution are detailed as follows:

1. According to the regulations published by the public security administration bureau of public security in 2008, “approval and reply of issues related to the project of gender-change in citizen's household registration after undergoing SRS,” the acceptance of such kind of cases by the judicial appraisal institution is compliant with the applicable national regulations.
2. According to the data sourced from the entrusting party, it is reaffirmed that Xiaoming was a male and has undergone the SRS, from male to female. A forensic examination was conducted to identify the female secondary sexual characteristics, including female breast, labium majus, minus, and vagina.
3. For humans, their gender identity can be classified into genetic gender, chromosomal gender, gonadal gender, genital gender, and psychological gender. The identification of gender is associated with the gender role played in a specific living environment. Gender is frequently transferred and consolidated not only in the social system but also in the course of individual socialization, which reflects a gender relationship that is acquired and changeable.

The definitive appraisal opinions: Xiao Ming received the male-to-female SRS under no restrictive operation conditions. Currently, Xiao Ming is female. Finally, Xiao Ming submitted the appraisal opinions to the local police department of household registration, which led to a success in changing the gender identity shown on her household registration from male to female. Eventually, she was issued a new ID card. In this circumstance, the appraisal institution gave recognition to the female gender of Xiao Ming, given her psychological gender and genital gender. It was also recognized that gender could be transferred and consolidated through individual socialization. Most importantly, it demonstrated that the Chinese government has put in place a policy aimed to support those transpersons who have received SRS to start a new life with a new ID.

## Discussion

People are entitled to choose their own gender. What is inspiring is the legal recognition given to this. To the best of our knowledge, there have been a number of similar judicial appraisals made in China over recent years. Eventually, all appraises were granted the corresponding gender identification. Once the identification is issued by the judicial authentication department, transpersons can go through the gender-changing procedures with the police station in an instant, before getting granted new citizenship and having the same rights as other Chinese citizens do. Notably, the public security departments routinely recommend the permission of the judicial authentication department instead of the hospital. Moreover, all of the applicants change from male to female. However, the female-to-male transpersons may be reluctant to come under such public pressure.

At present, there are still limited precise statistics about transsexuals in China. Firstly, a large proportion of transpersons are unwilling to have exposure to the public and they may choose to live abroad. Secondly, the mainstream media in China refrains from covering such news. Moreover, it is practically difficult to calculate the percentage of transgender population, which is attributed in part to the unified concept of transpersons.

**Figure 1:**
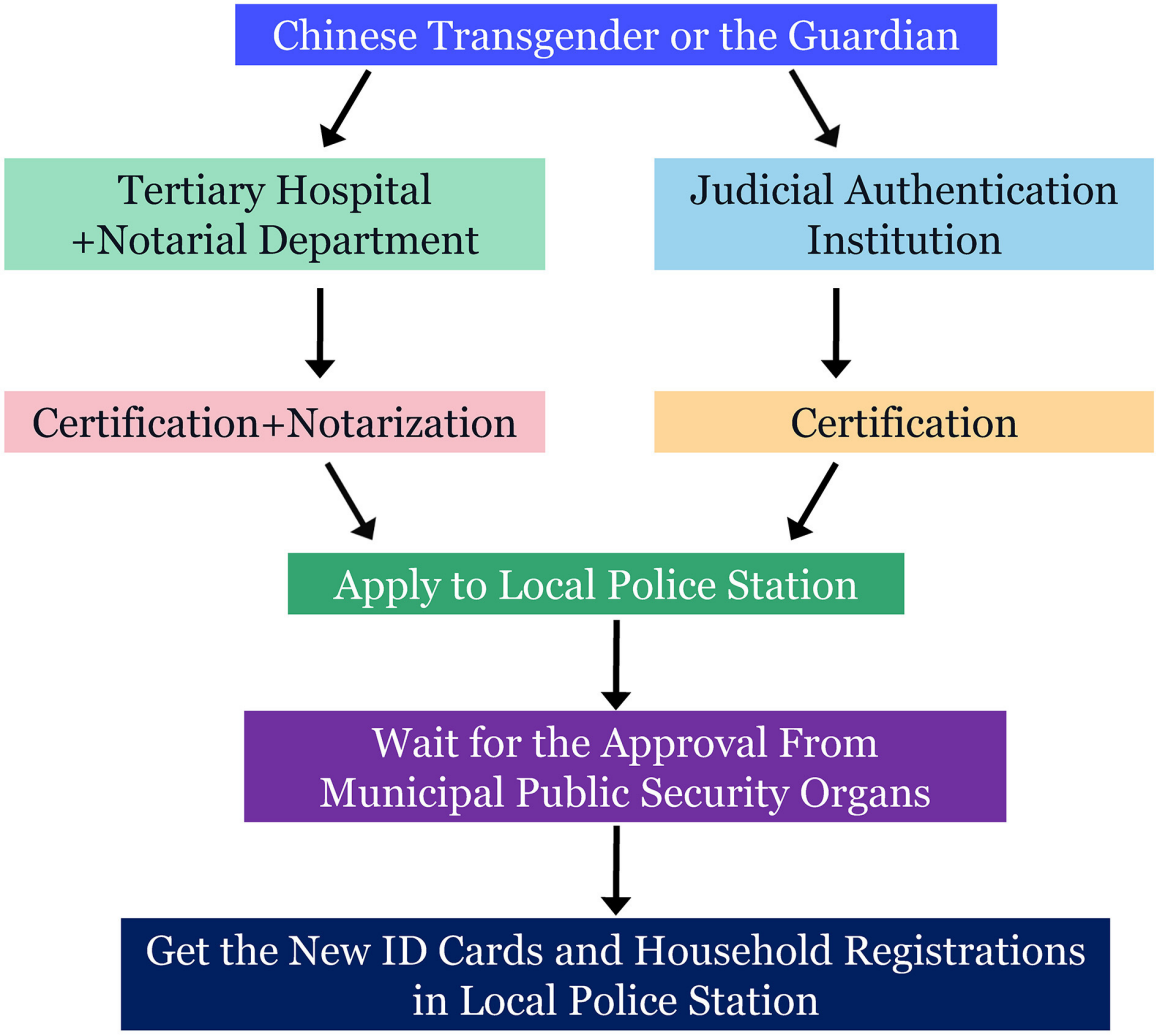
The detailed ID card change process for transpersons in China.

Despite a huge number of LGBTI in China, the process of protecting their rights through legislation remains at a very slow pace. In contrast, the social attitude toward LGBTI has improved in recent years. To a large extent, this is attributable to the efforts put in by many organizations and individuals over the years. As for the LGBTI civil engagement with a sense of mission, there has been remarkable achievements made in protecting the rights of homosexuals in China from various perspectives including law, policy, social and cultural attitude, etc. (11). In 2008, the policies aimed for household registration specifically involving transpersons after SRS were published by the Bureau of Public Security, which marks a key milestone in allowing those transpersons who received SRS to get a new ID card for starting a new life. The ID-changing process is detailed in Figure 1.

As a matter of fact, Chinese hospitals have no problem at all with performing SRS. However, there remain plenty of transpersons preferring Thailand or other countries for doing it, which is largely attributed to the complex procedures in China. For example, the requirements for surgery lasted for a minimum of 5 years without hesitation. It is a difficult period for transpersons who are keen on receiving SRS. Moreover, the psychological and psychiatric treatment carried out before the operation lasts at least 1 year. The applicants are required to be 18 or over and have received consent from their relatives with lineal consanguinity. Apart from that, there are many other rigid requirements. It is expected that, our Chinese policies will be made more considerate for transpersons in the future. Furthermore, marriage is possibly the most prominent right needing protection. So far, there are only a handful of countries or states around the world where recognition is given to same-sex marriage (12). Professor Yinhe Li, who is a researcher of the Chinese Academy of Social Sciences, is among the advocates for legalizing same-sex marriage. According to Li, there was a case occurring in 1997 when a gay soldier got caught having sex with another man and arrested as a rogue. After being detained, he received a certificate from the hospital stating that he was mentally ill. For this reason, homosexuality was not classified as a psychological anomaly until after 2001. “2001 was a turning point when there was the first-ever positive coverage of China's LGBTI community in the official media. After that, all official media outlets, whether paper, TV or online, started mentioning the LGBTI community a lot more.” With the elapse of nearly two decades, however, there remains a long way to go for the legalization of same-sex marriage in China. It is thus concluded that transgender marriage is less likely to be legally recognized. In spite of the little progress at present in China toward removing the discrimination and prejudice on transpersons, we found that transpersons with new identities have become more confident and happy in life in our limited interviews on Chinese transpersons for whom the SRS was performed. In the past, many transpersons in China were very nervous talking about their true sexual identities. But nowadays, more people are open and ready to let others know about them and fight for their rights. The current ID policy of transpersons, to some extent, is really making changes to a relatively inactive social environment. Meanwhile, it's important to praise equal rights between male and female, sexual majority and sexual minority, and revise the policy more inclusive and equal. The entitlements for transpersons are also very important like the gender affirmative ID and individualized medical security project.

Jin Xing has honestly chosen to be herself and active on the screen with her commendable personal abilities, without nothing concealed, which makes it inevitable for her to be targeted by some vicious cyber-violence. On the contrary, respect, and admiration are received from the audience. It is extremely tough to resolutely choose to be yourself when realizing that you are “not gregarious.” Finally, she responded by saying, “This stone is my choice, without anyone forcing me, and I want to stand on that stone.” (“I Can I BB” Qipa Shuo/Season 2, 20150711, iQIYI, Inc.). When the legal system in China improves constantly, there are more people who will feel safe after making attempt to maintain a balance on the stone. Moreover, those standing on the stone or the island can enjoy the same rights as their fellow citizens standing on the land.

## Data Availability Statement

The original contributions presented in the study are included in the article/supplementary material, further inquiries can be directed to the corresponding author/s.

## Author Contributions

ZL and JS: conceptualization, formal analysis, writing–original draft, and writing-review and editing. JC: conceptualization, formal analysis, writing–review and editing, funding acquisition, and project administration. MC and YG: writing-final draft and methodology. All authors contributed to the article and approved the submitted version.

## Funding

This work was supported by the Key Program of National Natural Science Foundation of China (No. 82030058) and the Excellent Postdoctoral Program for Innovative Talent of Hunan (2020RC2015).

## Conflict of Interest

The authors declare that the research was conducted in the absence of any commercial or financial relationships that could be construed as a potential conflict of interest.

## Publisher's Note

All claims expressed in this article are solely those of the authors and do not necessarily represent those of their affiliated organizations, or those of the publisher, the editors and the reviewers. Any product that may be evaluated in this article, or claim that may be made by its manufacturer, is not guaranteed or endorsed by the publisher.
